# Glutathione Peroxidase 4 Is a Predictor of Diabetic Kidney Disease Progression in Type 2 Diabetes Mellitus

**DOI:** 10.1155/2022/2948248

**Published:** 2022-10-12

**Authors:** Yi-hui Wang, Dong-yuan Chang, Ming-hui Zhao, Min Chen

**Affiliations:** ^1^Renal Division, Department of Medicine, Peking University First Hospital, Institute of Nephrology, Peking University, Key Laboratory of Renal Disease, Ministry of Health of China, Key Laboratory of Chronic Kidney Disease Prevention and Treatment (Peking University), Ministry of Education, Beijing 100034, China; ^2^Peking-Tsinghua Center for Life Sciences, Beijing, China; ^3^Research Units of Diagnosis and Treatment of Immune-Mediated Kidney Diseases, Chinese Academy of Medical Sciences, Beijing, China

## Abstract

**Background:**

Diabetic kidney disease (DKD) represents a heavy burden in type 2 diabetes mellitus (T2DM). Ferroptosis plays an important role in DKD, and it thus provides new perspectives to pursue more related biomarkers to assess the disease severity and prognosis. Glutathione peroxidase 4 (GPX4) is the mainstay in regulating ferroptosis. The current study investigated the predictive value of kidney GPX4 expression level in DKD progression.

**Methods:**

We measured GPX4 levels in kidney paraffin sections of 85 biopsy-proven DKD patients by immunohistochemistry staining. The associations between the GPX4 level and clinicopathological parameters as well as renal outcomes were analyzed.

**Results:**

GPX4 is mainly expressed in kidney tubulointerstitium, especially in tubular epithelial cells of DKD patients. The GPX4 expression level was significantly lower in DKD patients than healthy controls. Besides, GPX4 level significantly correlated with proteinuria (*r* = −0.42, *p* < 0.001), urinary albumin-to-creatinine ratio (uACR) (*r* = −0.40, *p* < 0.01), serum creatinine (Scr) (*r* = −0.59, *p* < 0.001), estimated glomerular filtration rate (eGFR) (*r* = 0.66, *p* < 0.001), and the percentage of sclerosed glomeruli (*r* = −0.42, *p* < 0.001) in renal specimens. During follow-up, the GPX4 level positively correlated with eGFR slope (*r* = 0.48, *p* < 0.001), and GPX4-low patients showed a significantly higher probability of developing end-stage kidney disease (ESKD) compared with GPX4-high patients (*p* < 0.01). Moreover, after adjusting for other potential predictors, the GPX4 level was still an independent predictor of developing ESKD (HR 2.15, 95% CI 1.08 to 4.28, *p* < 0.05).

**Conclusions:**

Kidney tubulointerstitial GPX4 expression level was associated with the disease severity and progression of DKD.

## 1. Introduction

The prevalence of diabetes mellitus (DM) is increasing steadily all over the world, and type 2 diabetes mellitus (T2DM) accounts for 90% of all DM patients [1, 2]. Diabetic kidney disease (DKD) is one of the most severe complications of DM and has become the leading cause of end-stage kidney disease (ESKD) [3–5]. Identifying patients at high risk of DKD progression in T2DM is critical for delaying or even preventing the disease progression. Despite extensive efforts to explore reliable predictors for kidney function decline, there are still only a limited number of prognostic biomarkers, including albuminuria, serum cystatin C, and baseline estimate glomerular filtration rate (eGFR) [6–9], as well as some pathological parameters including interstitial fibrosis and tubular atrophy (IFTA), interstitial inflammation, and vascular lesion score [10–12]. However, the known predictors for DKD progression have certain limitations [13, 14], including insufficient specificity and sensitivity of albuminuria for ESKD and estimation deviation of eGFR [15]. Moreover, albuminuria and eGFR largely reflect aspects of glomerular damage, while tubulointerstitium injury and structural damage are only partially captured by eGFR. Therefore, it is imperative to explore more perspective biomarkers for DKD progression.

Oxidative stress is one of the key aspects in the pathogenesis of DKD. Elevated prooxidant enzyme-induced reactive oxygen species (ROS) production together with insufficient antioxidants results in oxidative stress in DKD [16, 17]. Lipid peroxidation is an integral part of oxidative stress [18]. Ferroptosis, defined as iron- and lipid peroxidation-dependent novel form of regulated cell death (RCD) [19–21], was proved to participate in the pathogenesis of DKD [22–28]. Glutathione peroxidase 4 (GPX4), a unique intracellular antioxidant enzyme, is the key regulator of ferroptosis [29, 30]. GPX4 directly reduces phospholipid hydroperoxyl radical (PLOO•) and phospholipid hydroperoxide (PLOOH) into corresponding phospholipid alcohols (PLOH) to prevent chain propagation of lipid peroxidation and thus protects cells against lipid peroxidation in the membrane [19, 21, 31]. A genome-wide association study (GWAS) revealed that the GPX4 gene on the oxidative stress pathway may protect DKD progression [32]. Other studies also found that the GPX4 level was significantly decreased in kidneys of DKD mouse and in high-glucose-treated kidney tubular epithelial cells [22–24]. Therefore, it is of interest to investigate the association of kidney GPX4 expression and renal disease severity as well as outcomes in DKD patients.

## 2. Materials and Methods

### 2.1. Patients

A total of 85 patients with renal biopsy-proven DKD according to the criteria proposed by Renal Pathology Society [33] from January 2015 to December 2017 in Peking University First Hospital were enrolled in the study. None of these patients had coexisting nondiabetes-related renal disease (NDRD). All the patients were diagnosed with T2DM according to the criteria proposed by the American Diabetes Association in 2017 [34]. DKD was defined by the presence of suggestive glomerular lesions like nodular sclerosis, diffuse mesangial sclerosis, mesangial expansion, basement membrane thickening, arteriolar hyalinosis, microaneurysms, and exudative lesions [33]. Twenty diabetic patients with the biopsy-proven minimal change disease (MCD) and without DKD were recruited as disease control. Kidney samples of healthy controls (*n* = 14) were obtained from the healthy kidney poles of individuals receiving tumor nephrectomies. These healthy controls did not have a history of hypertension, diabetes, cardiovascular diseases, or renal diseases. All kidney samples of diabetic patients with MCD and without DKD and healthy controls were confirmed by pathological examinations including direct immunofluorescence, light microscopy, and electron microscopy. The investigation was conducted according to the Declaration of Helsinki and was approved by the Ethics Committee of Peking University First Hospital (2017-1280). Written informed consent was obtained from each participant.

### 2.2. Clinical and Laboratory Data

Clinical and laboratory data of these patients at the time of renal biopsy and follow-up were systematically recorded, including age, sex, diabetes history, proteinuria, urinary albumin-to-creatinine ratio (uACR), blood urea nitrogen (BUN), serum creatinine (Scr), eGFR, fasting blood glucose (FBG), hemoglobin A1c (HbA1c), hemoglobin (Hb), serum albumin, triglyceride (TG), high-density lipoprotein-cholesterol (HDL-C), and low-density lipoprotein-cholesterol (LDL-C). The eGFR was calculated using the CKD-EPI equation [35]. The eGFR slope was calculated using a linear mixed-effects model described in SAS studio [36].

### 2.3. Renal Histopathology

Renal specimens were evaluated using direct immunofluorescence, light microscopy, and electron microscopy. Biopsies were scored independently by two experienced pathologists, respectively. Diabetic glomerulopathy was classified as class I through IV according to the criteria proposed by Renal Pathology Society [33]. IFTA scores were assessed semiquantitatively based on the proportion of the tubulointerstitial compartment affected (0, none; 1, <25%; 2, 25–50%; and 3, >50%). Interstitial inflammation levels were scored semiquantitatively (0, absent; 1, infiltration only in areas related to IFTA; and 2, infiltration in areas without IFTA), and vascular lesions were scored according to the presence of arteriolar hyalinosis (0, no arteriolar hyalinosis; 1, one arteriole with hyalinosis; and 2, more than one arteriole with hyalinosis) and large-vessel arteriosclerosis (0, no intimal thickening is present; 1, intimal thickening is less than the thickness of the media; and 2, intimal thickening is more than the thickness of the media).

### 2.4. GPX4 Immunohistochemistry (IHC) Staining

Formalin-fixed paraffin-embedded kidney tissue sections were blocked by 3% bovine serum albumin after deparaffinization, rehydration, and heat-induced epitope retrieval and were stained with anti-GPX4 antibody (ab125066; Abcam; USA) overnight at 4°C. Subsequently, an HRP-DAB system (PV-9002; ZLI-9018; ZSBIO, Beijing, China) was used for color development. Twenty images were randomly taken for each section under 400x magnification in a blind fashion and quantified by Image-Pro Plus software V.6.0 (Media Cybernetics, Bethesda, MD).

### 2.5. Immunofluorescence Colocalization

Formalin-fixed paraffin-embedded kidney tissue sections were blocked after heat-induced epitope retrieval and stained with anti-GPX4 (ab125066; Abcam), anti-AQP1 (sc-25287; Santa Cruz, USA), anti-Calbindin D28k (sc-365360; Santa Cruz), anti-CD31 (sc-53411; Santa Cruz), anti-synaptopodin (ab259976; Abcam), and anti-integrin *α*8 (sc-365798; Santa Cruz) antibodies overnight at 4°C, respectively. Then, sections were incubated with Cy3-conjugated (A32727; Invitrogen, USA) or FITC-conjugated (A32731; Invitrogen) secondary antibodies and stained nucleus with DAPI (D9542; Sigma-Aldrich, USA). Images were taken under 400x magnification using Leica DM2500 microscope (Leica Microsystems, Germany).

### 2.6. Outcomes

ESKD, defined as an irreversible decline of kidney function (eGFR less than 15 mL/min/1.73 m^2^) leading to the need for a regular course of long-term dialysis or a kidney transplant to maintain life [37, 38], was employed as endpoint. The patients were followed up until 28 February 2021 or ESKD, whichever came first.

### 2.7. Statistical Analysis

Continuous data were presented as mean ± SD or median and interquartile range (IQR), as appropriate. Categorical data were described by absolute frequencies and percentages. Continuous clinical data were compared using Student's *t*-test or the Mann-Whitney *U* test as appropriate. The Pearson correlation analysis was performed to evaluate the association between immunohistochemistry staining intensity (IOD/area) and clinicopathological parameters.

The Kaplan-Meier analysis was used to assess the association between IOD/area and outcomes. The Cox proportional hazard regression was employed to evaluate the association between potential risk variables and outcomes. The analysis was performed with SPSS statistical software package (SPSS V.24.0; IBM) as well as survival, rms, timeROC, and survminer package in R studio (based on R x64 4.0.3). A *p* value less than 0.05 was considered statistically significant (^∗^*p* < 0.05, ^∗∗^*p* < 0.01, and ^∗∗∗^*p* < 0.001).

## 3. Results and Discussion

### 3.1. General Data

Among the 85 DKD patients, 64 were male and 21 were female, with an age of 48.6 ± 12.6 years at renal biopsy. The median duration of diabetes was 120 (60, 168) months. The proteinuria and uACR of these patients were 4.0 (2.3, 7.6) g/24 h and 3223.6 (1691.3, 5837.5) mg/g, respectively. The Scr and eGFR of these patients were 174.3 (105.0, 309.9) *μ*mol/L and 37.2 (20.3, 61.9) mL/min/1.73 m^2^, respectively. As for the pathological type, 5/85 (5.9%), 14/84 (16.5%), 60/85 (70.6%), and 6/85 (7.0%) of patients were categorized as class I, class II, class III, and class IV according to diabetic glomerulopathy, respectively, and 20/85 (23.5%), 24/85 (28.2%), and 41/85 (48.3%) of patients got IFTA scores as 1, 2, and 3, respectively. The detailed general data are listed in Table 1.

### 3.2. Association between GPX4 Levels and Clinicopathological Parameters

**Figure 1:**
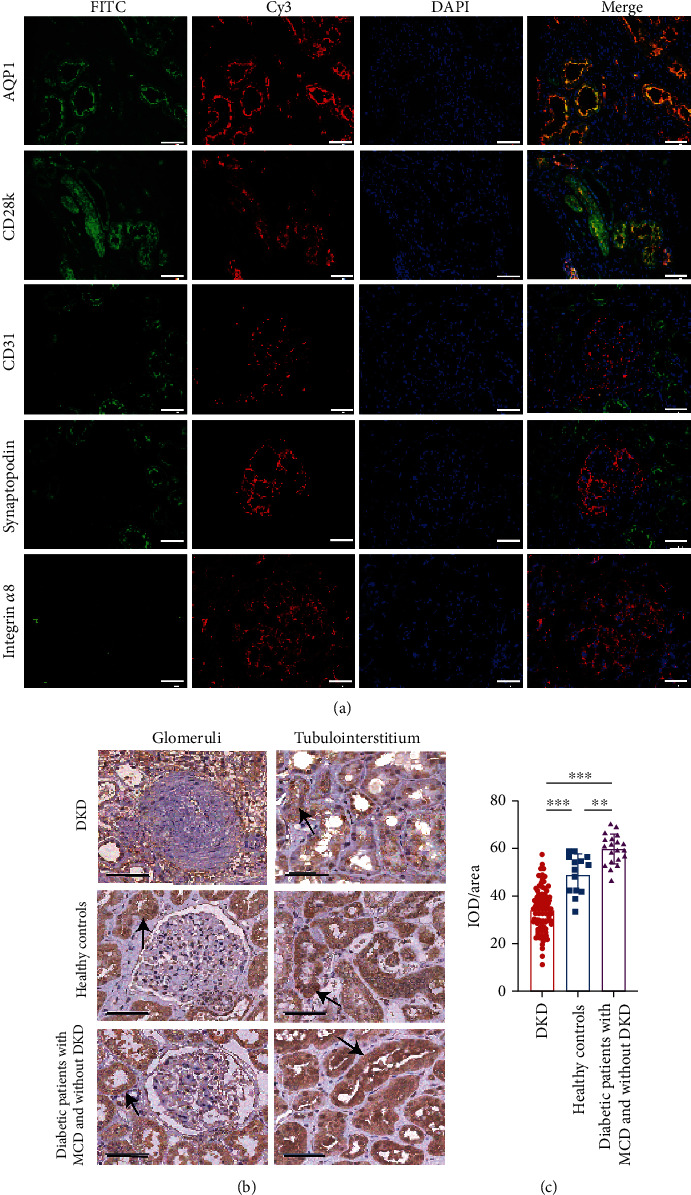
The expression of GPX4 in renal specimens. (a) GPX4 (marked with FITC-conjugated secondary antibody) mainly expressed in proximal and distal tubular epithelial cells (marked as AQP1 and CD28k with Cy3-conjugated secondary antibody, respectively), and there was little, if any, expression of GPX4 in glomerular podocytes and endothelial and mesangial cells (marked as synaptopodin, CD31, and integrin *α*8 with Cy3-conjugated secondary antibody, respectively). Renal specimens came from healthy controls. Scale bar = 50 *μ*m. (b, c) Representative photographs (b) and semiquantification (c) of GPX4 IHC staining in DKD patients (*n* = 85), MCD patients with T2DM (*n* = 20), and healthy controls (*n* = 14). The black arrow indicates the staining of GPX4, and the scale bar = 50 *μ*m (b). Horizontal lines represent mean ± SD (c). AQP1: Aquaporin 1; CD28k: Calbindin D28k; DKD: diabetic kidney disease; IOD/area: the staining intensity of GPX4; MCD: minimal change disease. ^∗^*p* < 0.05, ^∗∗^*p* < 0.01, and ^∗∗∗^*p* < 0.001.

IHC analysis of kidney slices was performed to evaluate GPX4 expression levels. In healthy controls, GPX4 was mainly expressed in kidney tubulointerstitium, especially in renal tubular epithelial cells (Figure 1(a)). The GPX4 level was significantly lower in DKD patients than that in healthy controls (34.6 ± 9.3 vs. 49.5 ± 8.3, *p* < 0.001) and diabetic patients with MCD and without DKD (34.6 ± 9.3 vs. 59.9 ± 6.3, *p* < 0.001) (Figures 1(b) and 1(c)). The detailed general data of healthy controls and diabetic patients with MCD and without DKD are listed in Table S1.

**Figure 2:**
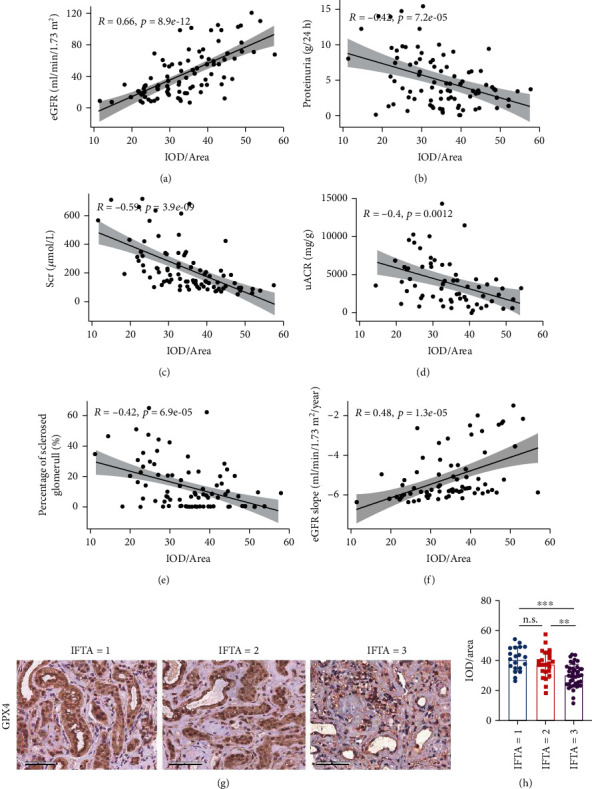
Correlations between GPX4 levels and clinicopathological parameters. (a–f) Kidney tubulointerstitial GPX4 level was significantly correlated with eGFR (a), proteinuria (b), Scr (c), uACR (d), percentage of sclerosed glomeruli (e), and eGFR slope (f). (g, h) Representative photographs (g) and semiquantification (h) of GPX4 IHC staining in DKD patients with different IFTA scores. Scale bar = 50 *μ*m (g). Horizontal lines represent mean ± SD (h). DKD: diabetic kidney disease; eGFR: estimated glomerular filtration rate; IFTA: interstitial fibrosis and tubular atrophy; IOD/area: the staining intensity of GPX4; Scr: serum creatinine; uACR: urinary albumin-to-creatinine ratio. ^∗^*p* < 0.05, ^∗∗^*p* < 0.01, and ^∗∗∗^*p* < 0.001.

Correlation analysis in DKD patients showed that the GPX4 level positively correlated with eGFR (*r* = 0.66, *p* < 0.001) and negatively correlated with proteinuria (*r* = −0.42, *p* < 0.001), uACR (*r* = −0.40, *p* < 0.01), Scr (*r* = −0.59, *p* < 0.001), and the percentage of sclerosed glomeruli in renal specimens (*r* = −0.42, *p* < 0.001) (Figures 2(a)–2(f)). Moreover, 9 out of 85 DKD patients had higher eGFR (defined as eGFR > 90 mL/min/1.73 m^2^) at kidney biopsy, which represents glomerular hyperfiltration of early DKD. The GPX4 level in the kidney was significantly higher in patients with higher eGFR (*n* = 9) than those patients with eGFR ≤ 90 mL/min/1.73 m^2^ (*n* = 76) (*p* < 0.01) (Figure S1). This was consistent with the positive correlation between GPX4 level and eGFR. The GPX4 level was significantly lower in DKD patients with higher IFTA score (IFTA score = 3) than those with lower IFTA score (IFTA score = 1, 2) (Figures 2(g) and 2(h)) and did not show significant difference between DKD patients with different scores of interstitial inflammation, arteriolar hyalinosis, or arteriosclerosis (Figure S2a-c).

### 3.3. Association between GPX4 Levels and Renal Outcomes

To investigate the association between the GPX4 level and renal outcomes, we performed time-dependent receiver operator curves (ROC, Figure S3). We used the Youden index of 5 years as the cut-off value of the GPX4 level (IOD/area = 37.28468), and 32/85 and 53/85 patients were classified as GPX4-high group and GPX4-low group, respectively. The clinical and laboratory parameters of these two groups are shown in Table S2. The median follow-up duration was 50 (44, 62) months, and the eGFR slope of patients was -5.8 (-6.0, -4.8) mL/min/1.73 m^2^/year. The eGFR slope was significantly higher in GPX4-high group than that in GPX4-low group (-5.5 (-5.9, -3.5) vs. -6.0 (-6.2, -5.6) mL/min/1.73 m^2^/year, *p* < 0.001); i.e., eGFR decreased more rapidly in GPX4-low group (Figure S4). Moreover, the GPX4 level positively correlated with the eGFR slope (*r* = 0.48, *p* < 0.001).

**Figure 3:**
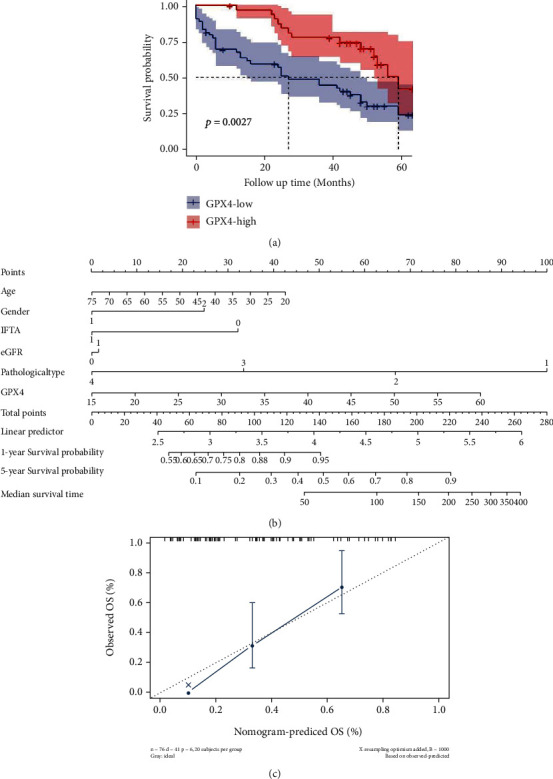
Association between GPX4 expression levels and renal outcomes. (a) Kaplan-Meier analysis of GPX4-high expression group and GPX4-low expression group. (b) Nomogram of multivariable Cox regression analysis. The length of each line segment reflected the contribution of this factor to the outcome events. Point referred to the single score corresponding to each variable under different values and the total score referred to the total of the single score corresponding to the values of all variables. Then, we could predict the survival probability and median survival time of patients based on the total scores. As the psm function in the rms R package did not accept 0 as the value of variables, the patients whose survival time was less than 2 months were removed, and finally, we got 76 patients left in the nomogram. (c) Calibration curve of nomogram. eGFR: estimated glomerular filtration rate; GPX4-high: high GPX4 expression in kidney tubulointerstitium; GPX4-low: low GPX4 expression in kidney tubulointerstitium; IFTA: interstitial fibrosis and tubular atrophy; OS: overall survival.

During the follow-up of 50 (44, 62) months, 50/85 (58.8%) patients progressed to ESKD. The Kaplan-Meier analysis showed that compared with patients in the GPX4-high group, those in the GPX4-low group had a significantly higher probability of developing ESKD (log-rank test, *p* < 0.01) (Figure 3(a)). In the multivariable Cox regression analysis, after adjusting for age, sex, IFTA score, pathological type, eGFR, and RAAS inhibitors use, the GPX4 level was still an independent factor associated with developing ESKD (HR 2.15, 95% CI 1.08 to 4.28, *p* < 0.05) (Table 2). Nomogram was employed to display the result of the Cox analysis (Figure 3(b)), and the GPX4 level was a valuable predictor as it had a long line. The accuracy of the nomogram was assessed by 20-sample-bootstrapped validation for the prediction of 5 years (Figure 3(c)). In addition, IFTA score was also an independent risk factor of developing ESKD (HR 2.75, 95% CI 1.14 to 6.63, *p* < 0.05) (Table 2).

Given that the time of inclusion of DKD patients spans 3 years, we also performed the multivariable Cox regression analysis three years after the diagnosis or ESKD as the outcome (whichever came first) to rule out the disturbance by the three-year time span. There are 39/85 endpoint events after a three-year follow-up. It was found that GPX4 level was still independently associated with developing ESKD (HR 2.73, 95% CI 1.20 to 6.19, *p* < 0.05) (Table S3). This was consistent with the above-mentioned results.

## 4. Discussion

A high glucose transport state in DKD exacerbates ROS production and results in oxidative stress [39, 40]. Previous studies found that ROS played a central role in ferroptosis [41, 42], and a microarray study found that the ferroptosis pathway was significantly enriched in the kidney tubulointerstitium of DKD patients [43]. GPX4 is the key regulator for ferroptosis and plays an antioxidant role in preventing lipid peroxidation. However, the association between GPX4 expression levels in kidney and disease severity as well as prognosis of DKD remains unclear.

In the current study, we found GPX4 mainly expressed in tubulointerstitium, especially in tubular epithelial cells, in renal specimens of DKD patients. Previous studies in animal models found that ferroptosis occurred in renal tubules in diabetes [22–24]. Since 60% of the oxygen consumption of kidneys is devoted to sodium reabsorption in proximal tubules to fulfill the high metabolic energy demand [44], high expression of GPX4 may act as an essential antioxidant force against massive ROS production in proximal tubular cells of DKD patients and defect antioxidant capacity in kidneys is a hallmark of DKD. Moreover, we found some associations between the GPX4 level and disease severity of DKD, including urinary protein, Scr, eGFR, and the percentage of sclerosed glomeruli in renal specimens. Patients with high GPX4 expression had better kidney outcome than those with low GPX4 expression. Meanwhile, the GPX4 level in tubulointerstitium was an independent predictor for renal outcomes, which supported the viewpoint that GPX4 play a protective role in the progression of DKD.

Consistent with our study, a previous study found that GPX4 mRNA level in kidney biopsy samples of diabetic patients was significantly lower than in nondiabetic patients, and tubular injury was significantly aggravated in GPX4-deficient mice, suggesting that GPX4 has a renal protective effect, especially in tubular cells [45]. A functional single nucleotide polymorphism (SNP) site of GPX4 (rs713041) was found to be associated with higher eGFR in French/Belgian patients and inversely associated with the prevalence of nephropathy in Brazilian patients [46]. In addition, previous studies showed higher circulating levels of malonaldehyde (MDA, the product of lipid peroxidation), iron, ferritin (biomarker for increased iron stores), and lower glutathione (GSH) levels in diabetic patients than in healthy controls [47–51], which suggested that lipid peroxidation, iron overload, and GSH deprivation occurred in diabetic patients, resulting in ferroptosis and consequently exacerbating diabetic complications. Therefore, as a ferroptosis regulator, GPX4 could provide a potential strategy for DKD prognosis assessment as a ferroptosis regulator. Moreover, some chemical compounds including glabridin [52], N-acetylcysteine [53], and platycodin D [54] could also significantly alleviate ferroptosis in DKD via maintaining redox homeostasis through activating GPX4, which may be inspiring for clinical treatment.

Nuclear factor erythroid 2-related factor 2 (NRF2) was a transcription factor of various antioxidant genes including GPX4, and it is known to suppress oxidative stress and ferroptosis [55–57]. Some studies found that activating NRF2 can significantly improve renal damage and inhibit ferroptosis in mice by upregulating GPX4 [23, 58], which suggested that ferroptosis, specially NRF2/GPX4 axis, played an important role in the progression of DKD. It is of interest to further investigate the role of NRF2/GPX4 axis in the development of DKD.

There were some limitations in our study. The sample size of the current study was limited, and most patients enrolled in this study were advanced DKD. Moreover, the pathogenic mechanism of DKD was complicated, and whether these results could be extrapolated to a broader patient population remains further investigation.

## 5. Conclusions

In conclusion, the current study showed that a lower GPX4 level was associated with more severe kidney disease and a higher risk of progression to ESKD in patients with DKD.

## Figures and Tables

**Table 1:** General data of DKD patients.

Parameters	
Number	85
Age (years)	48.6 ± 12.6
Sex, male/female	64/21
Diabetes history (months)	120 (60, 168)
Proteinuria (g/24 h)	4.0 (2.3, 7.6)
uACR (mg/g)	3223.6 (1691.3, 5837.5)
Scr (*μ*mol/L)	174.3 (105.0, 309.9)
BUN (mmol/L)	11.2 (7.7, 15.4)
eGFR (mL/min/1.73 m^2^)	37.2 (20.3, 61.9)
eGFR slope (mL/min/1.73 m^2^/year)	-5.8 (-6.0, -4.8)
FBG, mmol/L	6.5 (5.5,8.5)
HbA1c (%)	6.8 (5.8, 8.0)
Hb (g/L)	111.0 ± 23.2
Serum albumin (g/L)	32.8 ± 6.0
TG (mmol/L)	1.7 (1.2, 2.6)
HDL-C (mmol/L)	1.0 (0.8, 1.2)
LDL-C (mmol/L)	2.8 (2.0, 3.7)
Pathological type, I/II/III/IV	5/14/60/6
IFTA score, grade 0/1/2/3	0/20/24/41
Inflammation score, grade 0/1/2	0/33/52
Arteriole hyalinosis score, grade 0/1/2	22/63/0
Atherosclerosis score, grade 0/1/2	3/49/33
RAAS inhibitors (%)	70.6 (60/85)
Lipid-lowering drugs (%)	57.6 (49/85)

BUN: blood urea nitrogen; DKD: diabetic kidney disease; eGFR: estimated glomerular filtration rate; FBG: fasting blood glucose; Hb: hemoglobin; HbA1c: hemoglobin A1c; HDL-C: high-density lipoprotein-cholesterol; IFTA: interstitial fibrosis and tubular atrophy; LDL-C: low-density lipoprotein-cholesterol; RAAS: renin-angiotensin-aldosterone system; Scr: serum creatinine; TG: triglyceride; uACR: urinary albumin-to-creatinine ratio. The continuous data were expressed as mean ± SD or median and IQR as appropriate.

**Table 2:** Cox regression analysis for the endpoint in patients with DKD.

Factors	Univariable		Multivariable	
	HR (95% CI)	*p* value	HR (95% CI)	*p* value
Age	1.02 (0.99 to 1.04)	0.153	1.02 (1.00 to 1.05)	0.089
Sex (female vs. male)	0.38 (0.18 to 0.82)	0.013	0.41 (0.18 to 0.95)	0.037
IOD/area (GPX4-low vs. GPX4-high)	2.47 (1.33 to 4.60)	0.004	2.15 (1.08 to 4.28)	0.030
eGFR (>60 vs. ≤60 mL/min/1.73 m^2^)	0.34 (0.16 to 0.70)	0.004	1.11 (0.40 to 3.13)	0.841
IFTA (>2 vs. ≤2)	3.14 (1.74 to 5.69)	<0.001	2.75 (1.14 to 6.63)	0.024
Pathological type (I and II vs. III and IV)	1.77 (0.83 to 3.78)	0.142	1.43 (0.62 to 3.33)	0.402
RAAS inhibitors use (yes vs. no)	0.54 (0.30 to 0.97)	0.038	0.78 (0.41 to 1.49)	0.452

DKD: diabetic kidney disease; eGFR: estimated glomerular filtration rate; IFTA: interstitial fibrosis and tubular atrophy; IOD/area: the staining intensity of GPX4; RAAS: renin-angiotensin-aldosterone system.

## Data Availability

Data are available upon request.

## Supplementary Materials

Supplementary Materials Table S1: general data of healthy controls and disease controls. Table S2: comparison between GPX4-high and GPX4-low groups. Table S3: Cox regression analysis for the endpoint in patients with DKD using 3 years after the diagnosis or ESKD as outcome. Figure S1: GPX4 expression in DKD patients with higher eGFR and DKD patients with lower eGFR. Figure S2: GPX4 expression in DKD patients with different scores of interstitial inflammation, arteriolar hyalinosis, or arteriosclerosis. Figure S3: time-dependent ROC of GPX4 expression in DKD patients. Figure S4: eGFR slope in GPX4-high and GPX4-low groups.
