# Supplementary material for: Glutathione Peroxidase 4 Is a Predictor of Diabetic Kidney Disease Progression in Type 2 Diabetes Mellitus
Source: Oxid Med Cell Longev. 2022 Oct 12;2022:2948248. doi: 10.1155/2022/2948248 (PMC9581693; doi:10.1155/2022/2948248)

Table S1. General data of healthy controls and diseases controls

Parameters	Healthy controls	Diabetic patients with MCD and without DKD
Number of people	14	20
Age, years	48.6 ± 12.6	53.6 ± 14.6
Sex, Male/Female	11/3	6/14
Proteinuria, g/24h	-	3.7 (1.5-8.2)
Scr, μmol/L	88.6 (77.6-107.7)	80.4 (66.5-130.0)
BUN, mmol/L	5.5 ± 0.8	8.2 ± 5.7
eGFR, ml/min/1.73m^2	100.4 (79.0-114.4)	74.9 (61.4-102.9)
FBG, mmol/L	5.7 (5.3-6.3)	8.1 (7-8.6)
TG, mmol/L	1.4 (1.3-1.8)	5.2 (3.4-9.2)
Serum albumin, g/L	43.9 (40.0-47.9)	31.7 (18.7-43.6)

BUN, blood urea nitrogen; DKD, diabetic kidney disease; eGFR, estimated glomerular filtration rate; FBG, fasting blood glucose; MCD, minimal change disease; Scr, serum creatinine; TG, triglyceride. The continuous data were expressed as mean and standard deviation (SD) or median and interquartile range (IQR) as appropriate.

Table S2. Comparison between GPX4-high and GPX4-low group

Parameters	GPX4-high (n=32)	GPX4-low (n=53)
Proteinuria, g/24h**	3.2 (1.4-4.3)	6.0 (2.5-8.6)
uACR, mg/g***	1775.0 (881.8-3223.6)	4275.5 (2305.0-6372.5)
Scr, μmol/L***	105.5 (85.1-174.1)	234.2 (136.0-341.1)
BUN, mmol/L***	7.8 (5.8-10.6)	14.0 (9.7-21.8)
eGFR, ml/min/1.73m^2***	63.6 (41.6-84.2)	25.5 (14.2-47.6)
eGFR slope, ml/min/1.73m^2/year***	-5.5 (-5.9 - -3.5)	-6.0 (-6.2 - -5.6)
FBG, mmol/L	6.5 (5.6-11.3)	6.5 (5.3-8.2)
HbA1c, % *	7.8 (5.8-8.7)	6.4 (5.7-7.7)
TG, mmol/L	1.5 (1.2-2.6)	1.7 (1.2-2.7)
HDL-C, mmol/L	1.0 (0.8-1.4)	0.9 (0.8-1.1)
LDL-C, mmol/L	2.6 (2.0-3.5)	3.0 (2.1-3.7)
Serum albumin, g/L	33.8 (5.9)	32.1 (6.0)
Hb, g/L***	122.2 (22.8)	104.1 (21.0)
Percentage of sclerosed glomeruli, %*	6.0 (0-10.5)	10 (0-26.3)
Pathological type, I/II/III/IV	5/6/20/1	0/8/40/5
IFTA score, grade 0/1/2/3	0/13/12/7	0/7/12/34
Inflammation score, grade 0/1/2	0/15/17	0/18/35
Arteriole hyalinosis score, grade 0/1/2	8/24/0	14/39/0
Atherosclerosis score, grade 0/1/2	1/22/9	2/27/24
RAAS inhibitors, %**	87.5 (28/32)	60.4 (32/53)
Lipid-lowering drugs, %	50.0 (16/32)	62.3 (33/53)

BUN, blood urea nitrogen; DKD, diabetic kidney disease; eGFR, estimated glomerular filtration rate; FBG, fasting blood glucose; Hb, hemoglobin; HbA1c, hemoglobin A1c; HDL-C, high-density lipoprotein-cholesterol; IFTA, interstitial fibrosis and tubular atrophy; LDL-C, low-density lipoprotein-cholesterol; RAAS, renin-angiotensin-aldosterone system; Scr, serum creatinine; TG, triglyceride; uACR, urinary albumin-

to-creatinine ratio. *$p<0.05$, **$p<0.01$, ***$p<0.001$. The continuous data were expressed as mean and standard deviation (SD) or median and interquartile range (IQR) as appropriate.

Table S3. Cox regression analysis for the endpoint in patients with DKD using 3 years after the diagnosis or ESKD as outcome

Factors	Multivariable	
	HR (95%CI)	*p*-value
Age	1.02 (0.99 to 1.05)	0.138
Sex (Female vs. male)	0.56 (0.22 to 1.43)	0.224
IOD/area (GPX4-low vs. GPX4-high)	2.73 (1.20 to 6.19)	0.016
eGFR (>60 vs. ≤60 ml/min/1.73m^2)	2.51 (0.82 to 7.71)	0.109
IFTA (>2 vs. ≤2)	3.83 (1.39 to 10.56)	0.010
Pathological type (I & II vs. III & IV)	1.47 (0.60 to 3.59)	0.399
RAAS inhibitors use (yes vs. no)	0.69 (0.33 to 1.43)	0.317

DKD, diabetic kidney disease; eGFR, estimated glomerular filtration rate; IFTA, interstitial fibrosis and tubular atrophy; IOD/area, the staining intensity of GPX4; RAAS, renin-angiotensin-aldosterone system.

Figure S1. GPX4 expression in DKD patients with higher eGFR and DKD patients lower eGFR. Horizontal lines represent mean ± SD. IOD/area, the staining intensity of GPX4. **p*<0.05, ***p*<0.01, ****p*<0.001.

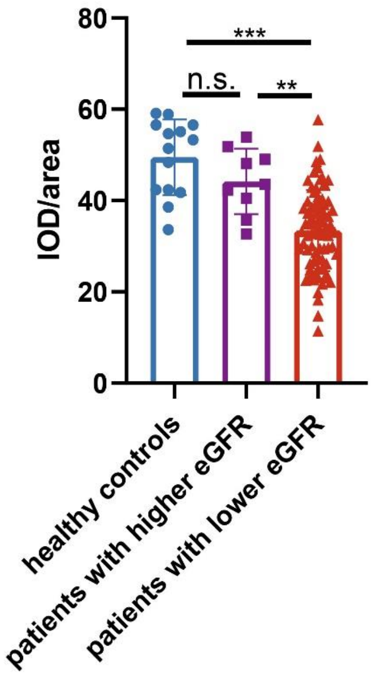

Figure S2. GPX4 expression in DKD patients with different score of interstitial inflammation, arteriolar hyalinosis or arteriosclerosis. **(a-c).** semi-quantification of GPX4 IHC staining in DKD patients (n=85) with different score of interstitial inflammation **(a)**, arteriolar hyalinosis **(b)** or arteriosclerosis **(c)**. Horizontal lines represent mean ± SD. IOD/area, the staining intensity of GPX4.

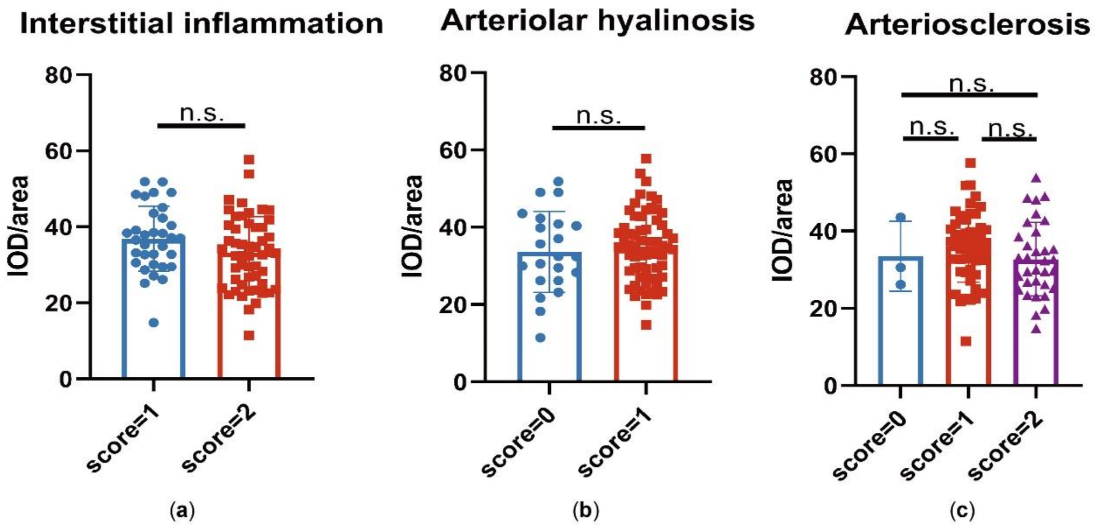

Figure S3. Time-dependent ROC of GPX4 expression in DKD patients.

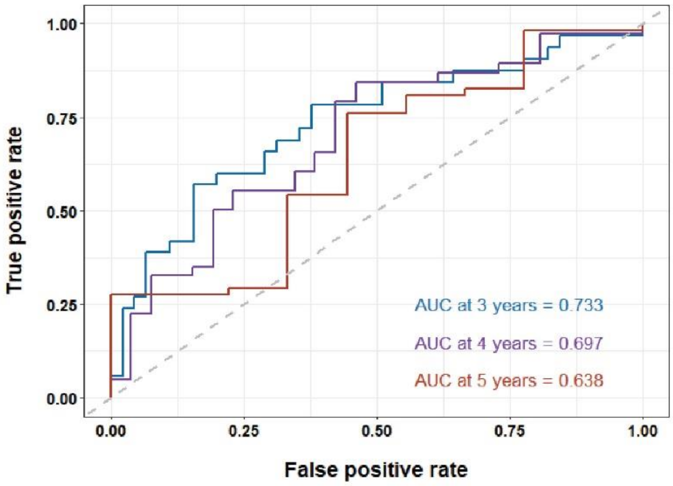

Figure S4. eGFR slope in GPX4-high and GPX4-low group. Horizontal lines represent mean ± SD. IOD/area, the staining intensity of GPX4. *$p<0.05$, **$p<0.01$, ***$p<0.001$.

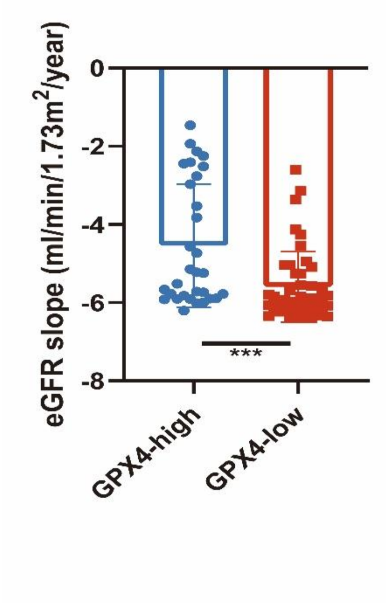

Supplement: Supplementary Materials — Table S1: general data of healthy controls and disease controls. Table S2: comparison between GPX4-high and GPX4-low groups. Table S3: Cox regression analysis for the endpoint in patients with DKD using 3 years after the diagnosis or ESKD as outcome. Figure S1: GPX4 expression in DKD patients with higher eGFR and DKD patients with lower eGFR. Figure S2: GPX4 expression in DKD patients with different scores of interstitial inflammation, arteriolar hyalinosis, or arteriosclerosis. Figure S3: time-dependent ROC of GPX4 expression in DKD patients. Figure S4: eGFR slope in GPX4-high and GPX4-low groups. [file 2948248.f1.pdf]
